# Association of State Share of Nonphysician Practitioners With Diagnostic Imaging Ordering Among Emergency Department Visits for Medicare Beneficiaries

**DOI:** 10.1001/jamanetworkopen.2022.41297

**Published:** 2022-11-10

**Authors:** Eric W. Christensen, Chi-Mei Liu, Richard Duszak, Joshua A. Hirsch, Timothy L. Swan, Elizabeth Y. Rula

**Affiliations:** 1 Harvey L. Neiman Health Policy Institute, Reston, Virginia; 2 Health Services Management, University of Minnesota, St Paul; 3 Department of Radiology, University of Mississippi Medical Center, Jackson; 4 Massachusetts General Hospital, Harvard Medical School, Boston, Massachusetts; 5 Marshfield Clinic, Marshfield, Wisconsin

## Abstract

**Importance:**

The use of nonphysician practitioners (NPPs) in the emergency department (ED) continues to expand, yet little is known about associations between NPPs and ED imaging use.

**Objective:**

To investigate whether the state share of ED visits for which an NPP was the clinician of record is associated with imaging studies ordered, given that state NPP share is associated with state-level NPP scopes of practice.

**Design, Setting, and Participants:**

This cross-sectional study compared diagnostic imaging ordering patterns associated with ED visits based on 2005-2020 Medicare claims for a nationally representative 5% sample of fee-for-service beneficiaries. For all 50 states and the District of Columbia, the state NPP share of ED visits by year was used to represent state-specific practice patterns for NPPs and physicians and how those patterns have evolved over time. The analysis controlled for patient demographic characteristics, Charlson Comorbidity Index scores, ED visit severity, year, and principal diagnosis.

**Exposures:**

The share of ED visits in each state in each year (state share) for which an NPP was the evaluation and management clinician.

**Main Outcomes and Measures:**

The main outcomes were the number and modality of imaging studies associated with ED visits. Analyses were by logistic regression and generalized linear model with γ-distribution and log-link function.

**Results:**

Among 16 922 274 ED visits, 60.0% involved women, and patients’ mean (SD) age was 70.3 (16.1) years. The share of all ED visits with an NPP as the clinician increased from 6.1% in 2005 to 16.6% in 2020. Compared with no NPPs, the presence of NPPs in the ED was associated with 5.3% (95% CI, 5.1%-5.5%) more imaging studies per ED visit, including a 3.4% (95% CI, 3.2%-3.5%) greater likelihood of any imaging order per ED visit and 2.2% (95% CI, 2.0%-2.3%) more imaging studies ordered per visit involving imaging.

**Conclusions and Relevance:**

In this study, use of NPPs in the ED was associated with higher imaging use compared with the use of only physicians in the ED. Although expanded use of NPPs in the ED may improve patient access, the costs and radiation exposure associated with more imaging warrants additional study.

## Key Points

**Question:**

Are emergency department (ED) nonphysician practitioner (NPP) encounters associated with more imaging studies than physician encounters?

**Findings:**

In this cross-sectional study of 16 922 274 ED visits by Medicare fee-for-service beneficiaries in 2005-2020, the presence of NPPs in the ED compared with no NPPs was associated with 5.3% more imaging studies per ED visit.

**Meaning:**

Use of NPPs in the ED setting appears to be associated with higher imaging use.

## Teaser

This cross-sectional study evaluates differences in diagnostic imaging ordering patterns for Medicare patients seen in emergency departments by nonphysician practitioners vs physicians.

## Introduction

In 2019, there were 151 million emergency department (ED) visits in the US.^1^ Although ED visits per 1000 people have been relatively stable in recent years (420 in 2009 to 439 in 2018),^2^ this rate has increased over time (eg, 353 in 1997 and 391 in 2007).^3^ Multiple studies have also shown increased use of diagnostic imaging in the ED over time. Of note, between 2004 and 2014, imaging studies per ED visit increased 29%.^4^ Overall, the use of ED imaging has increased for all modalities but more for computed tomography (CT), magnetic resonance imaging (MRI), and ultrasound than radiography; in terms of volume, much of the increase in ED imaging has been due to CT.^4,5,6,7,8,9,10,11,12,13,14^

Like other practice settings, the ED has seen substantial growth in nonphysician practitioners (NPPs), including nurse practitioners and physician assistants. In 1997, 5.2 million ED visits involved an NPP vs 15.2 million in 2006.^15^ More recent data have shown a continuation of this trend, with 22.5 million and 35.3 million ED visits involving an NPP in 2010 and 2018, respectively.^16,17^ In the outpatient office setting, NPP encounters have been associated with higher use of imaging than physician encounters.^18^ One study that compared imaging use in EDs staffed with NPPs with those without NPPs found that EDs with NPPs had 11% more imaging per ED visit.^19^ However, the study was not able to control for patient- and visit-level factors, such as demographic characteristics, comorbidities, diagnoses, and visit severity, given that the unit of analysis was the ED rather than the ED visit.

Laws governing the autonomy or physician oversight of NPPs vary by state and, over time, have generally increased scope of practice and/or autonomy for NPPs.^20^ In states with an increased scope of practice, there has been more growth in the number of NPPs as well as increased access to and delivery of health care services by NPPs.^21,22,23^

Given both the increased use of diagnostic imaging studies in the ED and the increased delivery of care in EDs by NPPs, we sought to understand how ED imaging ordering patterns differ on the basis of the share of ED visits managed by NPPs. Hypothesizing that a higher NPP share would be associated with both a greater likelihood of imaging during the ED visit and a greater number of imaging studies when a visit has imaging, we evaluated differences in ED imaging ordering patterns based on the share of ED visits in each state by year for which the clinician of record was an NPP rather than a physician.

## Methods

### Data Sources

This cross-sectional study used a Medicare 5% Research Identifiable File from 2005 to 2020 from the Centers for Medicare & Medicaid Services as the primary data source. These data include patient-level claims information and are a nationally representative sample of Medicare fee-for-service beneficiaries. On the basis of beneficiary zip code, we classified a beneficiary’s residence as metropolitan or not using rural-urban commuting area codes.

### Study Population

Our analysis included all ED visits from 2005 to 2020 at EDs in the 50 states and the District of Columbia (DC) for patients residing in the 50 states or DC. We restricted our analysis to ED visits for which the clinician submitting the evaluation and management claim (*Current Procedural Terminology* codes 99281-99285) was an emergency medicine, family practice, or internal medicine physician or nurse practitioner or physician assistant as designated in the Medicare data. We excluded ED visits for which the diagnosis on the evaluation and management claim was related to pregnancy, childbirth, perinatal conditions, congenital malformations, and supplemental codes (*International Statistical Classification of Diseases, Tenth Revision, Clinical Modification *[*ICD-10-CM*] codes O00-Q99 and U00-Z99 and *International Classification of Diseases, Ninth Revision, Clinical Modification *codes 630-679, 740-779, V01-V91, and E000-E999). The Advarra institutional review board deemed this study exempt from oversight as it posed no or minimal risk to human participants. This study followed the Strengthening the Reporting of Observational Studies in Epidemiology (STROBE) reporting guideline.

### Outcomes

Our primary outcome was the number of imaging studies ordered during the ED visit. We identified imaging studies using Neiman Imaging Types of Service codes, which classify *Current Procedural Terminology* and Healthcare Common Procedure Coding System codes by imaging type.^24^ For each ED visit, we included all imaging claims with an ED place of service occurring on the day of the evaluation and management claim. In addition, because an ED visit might span midnight, we included the patient’s imaging the day before and day after the evaluation and management claim if the place of service was the ED. If the charge on the imaging claim was $0, we excluded it. Our secondary outcome was the number of imaging studies by modality, including CT, radiography/fluoroscopy (radiography), and other (MRI, ultrasound, etc, which were grouped together because of their small share of ED imaging). Note that the focus was on the imaging performed based on whether the evaluation and management clinician requesting the study was an NPP or a physician and not on the specialty of the clinician who interpreted the imaging.

### Covariates

The key variable of interest was the share of ED visits in each state in each year for which the evaluation and management clinician was an NPP, which we call state-year NPP share. We defined NPP share at the state level rather than for each specific visit because of state-specific scopes of practice and other variations in how NPPs practice in the ED. We considered the state-year NPP share an instrument or proxy for the likelihood that an individual ED visit was managed by an NPP. Furthermore, because claims data lack the granularity in patient and clinical data required to fully risk adjust by ED visit, use of the state-year NPP share avoids bias due to the endogeneity inherent in case severity and selection of clinician type for a specific ED visit.

Other covariates included the following patient-level variables: sex, age (<65, 65-69, 70-74, 75-79, 80-84, 85-89, and ≥90 years), Medicare-reported race and ethnicity as a control variable (Black, White, other [Asian, Hispanic, North American Native, other, and unknown]), Charlson Comorbidity Index score (1, 2, and ≥3), and metropolitan residence (vs nonmetropolitan, including micropolitan, small town, or rural). Emergency department visit–level variables included year, severity (on a 5-point scale of increasing severity of 1-3, 4, and 5), and diagnosis group by *ICD-10-CM* code (infections, A00-B99; neoplasms, C00-D49; hematologic, D50-D89; endocrine, metabolic, E00-E89; mental/behavioral disorder, F01-F99; nervous system including eye and ear, G00-H95; circulatory, I00-I99; respiratory, J00-J99; digestive, K00-K95; dermatologic, L00-L99; musculoskeletal, M00-M99; genitourinary, N00-N99; symptoms, signs, and abnormal findings, R00-R99; injury, poisoning, S00-T88). In addition, we controlled for patients with cancer, who died the day of the ED visit, who were admitted as an inpatient immediately after the ED encounter, or who had any claim in the preceding 30 days.

### Statistical Analysis

Because many ED visits do not involve imaging, we used a 2-stage model to analyze the association between NPP share and imaging ordering patterns given the heavy weight on 0 (ie, no imaging).^25^ Stage 1 used logistic regression to evaluate the association of NPP share with the likelihood of a patient receiving any imaging vs no imaging. For those with imaging, stage 2 used a generalized linear model with a γ-distribution and a log-link function to estimate the amount of imaging.^26^ We selected the γ-distribution on the basis of the Akaike information criterion. The total number of imaging studies is the product of stages 1 and 2.

To estimate the association of NPPs with imaging performed, we used the model results to predict the amount of imaging if the state-year NPP share of ED visits was 0. In this way, the imaging volume comparison is between the current NPP-physician mix and what it would be if the ED visits included only physicians. It is not a comparison between only NPPs vs only physicians.

We conducted these analyses for imaging overall and separately for CT, radiography, and other modalities. A sensitivity analysis was conducted to confirm robustness of results to potential biasing factors. Specifically, we limited the analysis to ED visits for which the patient did not have cancer, was not admitted to the hospital, did not die the same day, and did not have any claim in the preceding 30 days (sensitivity sample). Finally, we repeated this analysis for subsets of ED visits based on diagnosis group. For statistical significance, we used an α of .05 and 2-sided tests. We constructed the analytical file using SAS, version 9.4 (SAS Institute Inc) and performed all statistical analyses using Stata, version 17.0 (StataCorp LLC).

## Results

There were 16 922 274 ED visits that met inclusion criteria. Sixty percent of patients were women and 40.0% were men; the mean (SD) age was 70.3 (16.1) years; 15.3% of patients were Black, 78.5% were White, and 6.2% were of another race or ethnicity (Table 1). A total of 1 911 255 visits (11.3%) were attributed to an NPP. Patients whose ED clinician was an NPP rather than a physician were more likely to be younger, White, and living outside a metropolitan area and had fewer comorbidities and a lower visit severity. The share of all ED visits for which the evaluation and management clinician was an NPP was 6.1% in 2005; this share steadily rose each year to 16.6% in 2020 (Figure).

**Table 1.** Emergency Department (ED) Visit Patient Demographic Characteristics by Clinician Type^a^

Characteristic	No. (%)			Percentage of all ED visits per characteristic	
	All ED visits	NPPs	Physicians	NPPs	Physicians
All ED visits	16 922 274 (100)	1 911 255 (100)	15 011 019 (100)	11.3	88.7
Age, y					
<65	4 677 083 (27.6)	723 360 (37.8)	3 953 723 (26.3)	15.5	84.5
65-69	2 222 410 (13.1)	274 812 (14.4)	1 947 598 (13.0)	12.4	87.6
70-74	2 269 307 (13.4)	245 360 (12.8)	2 023 947 (13.5)	10.8	89.2
75-79	2 236 106 (13.2)	216 235 (11.3)	2 019 871 (13.5)	9.7	90.3
80-84	2 224 978 (13.2)	192 055 (10.0)	2 032 923 (13.5)	8.6	91.4
85-89	1 870 806 (11.1)	152 055 (8.0)	1 718 751 (11.4)	8.1	91.9
≥90	1 421 584 (8.4)	107 378 (5.6)	1 314 206 (8.8)	7.6	92.4
Sex					
Female	10 147 280 (60.0)	1 140 430 (59.7)	9 006 850 (60.0)	11.2	88.8
Male	6 774 994 (40.0)	770 825 (40.3)	6 004 169 (40.0)	11.4	88.6
Race and ethnicity					
Black	2 593 515 (15.3)	312 618 (16.4)	2 280 897 (15.2)	12.1	87.9
White	13 279 566 (78.5)	1 473 959 (77.1)	11 805 607 (78.6)	11.1	88.9
Other^b^	1 049 193 (6.2)	124 678 (6.5)	924 515 (6.2)	11.9	88.1
Residence					
Metropolitan	13 571 832 (80.2)	1 497 293 (78.3)	12 074 539 (80.4)	11.0	89.0
Nonmetropolitan	3 350 442 (19.8)	413 962 (21.7)	2 936 480 (19.6)	12.4	87.6
Charlson Comorbidity Index score					
0	4 156 781 (24.6)	550 477 (28.8)	3 606 304 (24.0)	13.2	86.8
1	3 083 475 (18.2)	375 930 (19.7)	2 707 545 (18.0)	12.2	87.8
2	2 371 304 (14.0)	261 843 (13.7)	2 109 461 (14.1)	11.0	89.0
≥3	6 875 619 (40.6)	662 413 (34.7)	6 213 206 (41.4)	9.6	90.4
Unknown	435 095 (2.6)	60 592 (3.2)	374 503 (2.5)	13.9	86.1
ED visit severity					
1-3	3 579 738 (21.2)	701 646 (36.7)	2 878 092 (19.2)	19.6	80.4
4	4 770 045 (28.2)	620 397 (32.5)	4 419 648 (27.6)	13.0	87.0
5	8 572 491 (50.7)	589 212 (30.8)	7 983 279 (53.2)	6.9	93.1

Abbreviation: NPP, nonphysician practitioner.

^a^ Source data were from the 2005-2020 Medicare 5% Research Identifiable File.

^b^ Other race and ethnicity included Asian, Hispanic, North American Native, other, and unknown.

**Figure.**
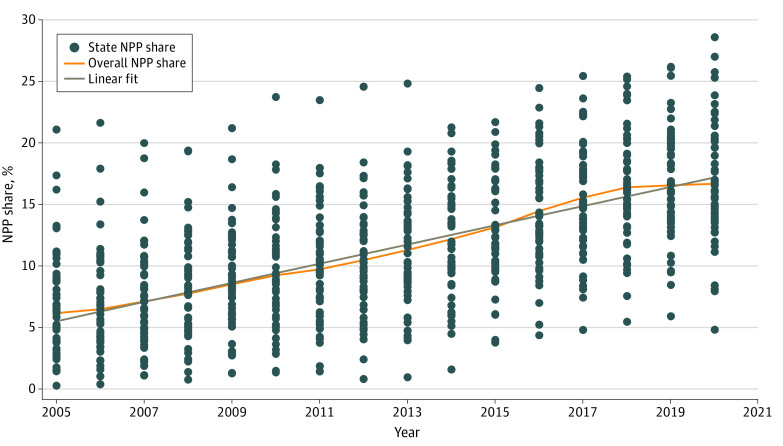
State and Overall Nonphysician Practitioner (NPP) Share by Year The graph shows the share of ED visits by state by year for which an NPP was the evaluation and management clinician.

Analysis of unadjusted means showed that 50% of all ED visits involved imaging and that for those visits with imaging, the mean (SD) number of imaging studies was 1.68 (1.13). Across all ED visits, the mean (SD) number of imaging studies was 0.84 (1.16) (Table 2). Specifically, the unadjusted mean number of imaging studies per ED visit was −0.04 less per visit with NPPs vs physicians (mean [SD] number of imaging studies, 0.80 [1.11] vs 0.84 [1.16]; *P* < .001).

**Table 2.** Imaging Values per Emergency Department (ED) Visit by Clinician Type

	All ED visits	NPPs	Physicians	Difference, %	*P* value
**Imaging studies per visit, mean (SD)**					
All modalities	0.84 (1.16)	0.80 (1.11)	0.84 (1.16)	−0.04 (−5.3)	<.001
CT	0.27 (0.63)	0.23 (0.58)	0.28 (0.63)	−0.05 (−16.8)	<.001
Radiography	0.51 (0.79)	0.52 (0.81)	0.51 (0.79)	0.01 (1.3)	<.001
Other modalities	0.05 (0.26)	0.05 (0.24)	0.05 (0.27)	0.00 (−8.9)	<.001
**Visits with imaging, % (SD)**					
All modalities	0.50 (0.50)	0.49 (0.50)	0.50 (0.50)	−0.01 (−2.8)	<.001
CT	0.20 (0.40)	0.17 (0.38)	0.21 (0.40)	−0.03 (−16.9)	<.001
Radiography	0.39 (0.49)	0.38 (0.49)	0.39 (0.49)	−0.01 (−3.1)	<.001
Other modalities	0.04 (0.20)	0.04 (0.20)	0.04 (0.20)	0.00 (−3.3)	<.001
**Imaging studies per visit for visits with imaging, mean (SD)**					
All modalities	1.68 (1.13)	1.64 (1.07)	1.68 (1.14)	−0.04 (−2.6)	<.001
CT	1.35 (0.71)	1.35 (0.68)	1.35 (0.71)	0.00 (0.1)	.14
Radiography	1.30 (0.76)	1.36 (0.78)	1.30 (0.75)	0.06 (4.6)	<.001
Other modalities	1.18 (0.51)	1.12 (0.40)	1.19 (0.52)	−0.07 (−5.8)	<.001

Abbreviations: CT, computed tomography; NPP, nonphysician practitioner.

Controlling for severity and other covariates, Table 3 shows the adjusted net increase in imaging studies using the 2-stage model to estimate the number of imaging studies if NPPs were not present in the ED (ie, 0% NPP share) compared with the observed mean NPP share of 11.3% over the study period. Across all ED visits with NPPs, the observed mean (SD) percentage of visits with imaging was 20.2% (0.4%) for CT, 39.3% (0.5%) for radiography, and 4.4% (0.2%) for other modalities. For imaging overall, state NPP share was associated with a 3.4% (95% CI, 3.2%-3.5%) higher likelihood of patients undergoing at least 1 imaging study. In addition, for those who underwent imaging, there was a 2.2% (95% CI, 2.0%-2.3%) increase in the number of studies associated with the presence of NPPs. Combined, there was a 5.3% (95% CI, 5.1%-5.5%) increase in imaging studies per ED visit given the presence of NPPs in the ED. By modality, the net increase on mean imaging studies was 7.3% (95% CI, 6.9%-7.7%) for CT, 3.2% (95% CI, 3.0%-3.5%) for radiography, and 14.2% (95% CI, 13.2%-15.2%) for other modalities. For the sensitivity sample, the changes overall (5.2%; 95% CI, 4.6%-5.7%) and by modality (CT, 7.6% [95% CI, 6.6%-8.6%]; radiography, 3.0% [95% CI, 2.4%-3.6%]; and other, 16.8% [95% CI, 14.3%-19.5%]) were consistent with the results for all ED visits. For the associations of patient and visit characteristics with imaging use, see eTables 1 and 2 in the Supplement. Specifically, patients who were older, were women, had comorbidities, or had higher visit severity were more likely to have imaging, whereas patients who were Black or of other race and ethnicity, died, or had other recent care were less likely to have imaging.

**Table 3.** Estimated Change in Imaging Counts per Emergency Department (ED) Visit Associated With the Presence of Nonphysician Practitioners (NPPs) by Modality^a^

	All	All ED visits			Sensitivity analysis			
		CT	Radiography	Other^b^	All	CT	Radiography	Other^b^
**Observed, mean (SD)**								
Had imaging, %	49.9 (50.0)	20.2 (40.1)	39.3 (48.8)	4.4 (20.5)	48.5 (50.0)	19.0 (39.3)	38.3 (48.6)	3.9 (19.2)
Mean No. of imaging studies for visits with imaging	1.677 (1.129)	1.352 (0.707)	1.303 (0.756)	1.183 (0.509)	1.674 (1.131)	1.358 (0.720)	1.327 (0.776)	1.197 (0.531)
Mean No. of imaging studies per visit	0.837 (1.157)	0.273 (0.629)	0.512 (0.793)	0.052 (0.265)	0.812 (1.149)	0.259 (0.619)	0.508 (0.804)	0.046 (0.253)
**Estimated (if NPP share is 0)**								
Had imaging, %	48.29	18.87	38.00	3.84	46.88	17.73	37.04	3.29
Mean No. of imaging studies for visits with imaging	1.641	1.347	1.303	1.182	1.642	1.355	1.327	1.197
Mean No. of imaging studies per visit	0.794	0.254	0.496	0.045	0.773	0.240	0.493	0.039
**Observed vs estimated**								
Had imaging	1.034	1.070	1.034	1.141	1.036	1.074	1.033	1.170
*P* value	<.001	<.001	<.001	<.001	<.001	<.001	<.001	<.001
Mean No. of imaging studies for visits with imaging	1.022	1.004	1.001	1.001	1.019	1.003	1.000	1.000
*P* value	<.001	<.001	.26	.33	<.001	.20	.40	.40
Mean No. of imaging studies per visit	1.053	1.073	1.032	1.142	1.052	1.076	1.030	1.168
*P* value	<.001	<.001	<.001	<.001	<.001	<.001	<.001	<.001

Abbreviation: CT, computed tomography.

^a^ The sensitivity analysis was limited to ED visits for which the patient did not have cancer, was not admitted to the hospital, died the same day, or had any claim in the 30 days prior.

^b^ For example, magnetic resonance imaging and ultrasound.

Table 4 shows the net increase in the number of imaging studies per ED visit associated with the presence of NPPs. By *ICD-10-CM* diagnosis group, these counts ranged from a low of 2.7% (95% CI, 2.2%-3.1%) more for injury or poisoning to a high of 11.7% (95% CI, 6.7%-17.3%) more for neoplasms. For every diagnosis group, there was a significant increase in the mean number of imaging studies per ED visit. These increases were associated with increases in both the likelihood of imaging and the number of imaging studies when imaging occurred. These findings were consistent with the sensitivity analysis.

**Table 4.** Estimated Change in Imaging Counts per Emergency Department (ED) Visit Associated With the Presence of Nonphysician Practitioners by *International Statistical Classification of Diseases, Tenth Revision, Clinical Modification* (*ICD-10-CM*) Diagnosis Group^a^

Description (*ICD-10-CM* group)	All ED visits						Sensitivity analysis					
	Had imaging		Mean No. of imaging studies for visits with imaging		Mean No. of imaging studies per visit		Had imaging		Mean No. of imaging studies for visits with imaging		Mean No. of imaging studies per visit	
	Change	*P* value	Change	*P* value	Change	*P* value	Change	*P* value	Change	*P* value	Change	*P* value
Infections (A00-B99)	1.043	<.001	1.029	<.001	1.073	<.001	1.064	.03	0.984	.29	1.046	.16
Neoplasms (C00-D49)	1.071	<.001	1.044	.007	1.117	<.001						
Hematologic (D50-D89)	1.060	<.001	1.004	.36	1.064	<.001	1.071	.09	1.019	.33	1.091	.08
Endocrine, metabolic (E00-E89)	1.086	<.001	1.023	<.001	1.110	<.001	1.080	<.001	1.022	.08	1.103	<.001
Mental/behavioral (F01-F99)	1.073	<.001	1.022	.001	1.095	<.001	1.105	<.001	1.024	.11	1.130	<.001
Nervous system (G00-H95)	1.034	<.001	1.028	<.001	1.062	<.001	1.042	.01	1.010	.25	1.051	.01
Circulatory (I00-I99)	1.016	<.001	1.024	<.001	1.041	<.001	1.021	.01	1.029	<.001	1.050	<.001
Respiratory (J00-J99)	1.018	<.001	1.011	<.001	1.029	<.001	1.021	.002	1.002	.37	1.023	.01
Digestive (K00-K95)	1.067	<.001	1.036	<.001	1.103	<.001	1.075	<.001	1.026	<.001	1.100	<.001
Dermatologic (L00-L99)	1.057	<.001	1.038	<.001	1.095	<.001	1.082	<.001	1.052	<.001	1.136	<.001
Musculoskeletal (M00-M99)	1.043	<.001	1.006	.021	1.048	<.001	1.049	<.001	1.001	.39	1.048	<.001
Genitourinary (N00-N99)	1.076	<.001	1.022	<.001	1.098	<.001	1.101	<.001	1.022	.01	1.122	<.001
Symptoms, signs, and abnormal findings (R00-R99)	1.033	<.001	1.022	<.001	1.056	<.001	1.037	<.001	1.024	<.001	1.061	<.001
Injury or poisoning (S00-T88)	1.017	<.001	1.010	<.001	1.027	<.001	1.012	.001	1.010	.01	1.022	<.001

^a^ The sensitivity analysis was limited to ED visits for which the patient did not have cancer, was not admitted to the hospital, died the same day, or had any claim in the 30 days prior.

## Discussion

In this cross-sectional study of nearly 17 million Medicare ED encounters over 16 years, we found that the presence of NPPs in the ED compared with no NPPs was associated with 5.3% more imaging studies during ED visits. Such differential use of imaging is not unique to the ED, as NPPs have been found to be 1.34 times more likely to order imaging than physicians in the outpatient office setting.^18^ Another primary care study of low-value back imaging found that nurse practitioners had higher imaging rates than physicians, but the difference was not significant.^27^

Our results are consistent with a previous study that investigated the association of NPPs in the ED with imaging use and found that EDs with NPPs had 11% more imaging per ED visit.^19^ Our results add credence to those findings by accounting for patient- and visit-level factors, such as demographic characteristics, comorbidities, diagnoses, and visit severity. In addition, incorporating the state-year NPP share of ED visits enabled us to distinguish and control for the level of NPP involvement given differences in the percentage of care delivered by NPPs across states and over time. Such differences may be associated with differences in NPP scopes of practice across states and over time along with other factors, such as ED team structure, incentive structure, and malpractice environment.

Three studies using the National Hospital Ambulatory Medical Care Survey showed fewer imaging studies for ED visits managed by NPPs vs physicians.^28,29,30^ However, 2 of those studies^28,29^ were descriptive only and did not control for patient and hospital characteristics. Although the third study^30^ included these controls, all 3 studies lacked the clinical data necessary to fully risk adjust ED visits to make a fair comparison of ED visits with NPPs vs physicians. Although the current study also lacked clinical data, we minimized the potential endogeneity challenges in modeling imaging ordering patterns based on the use of an NPP or physician for a specific ED visit by using the state-year shares of NPPs as an instrument.

Because the Medicare Physician Fee Schedule pays for services rendered by NPPs at 85% of what it pays physicians, NPPs deliver care at a lower cost if their use of concurrent and downstream services is the same as that of physicians. However, such a cost advantage is offset to the degree that NPPs are associated with higher resource use. Thus, our findings are important because they show a higher use of imaging studies across all modalities. Although this study did not focus on the cost question, observation of increased imaging use confirms that there is at least some offset. Future research should explore the magnitude of this offset and whether it partially or completely offsets the savings associated with the 85% rule.

Beyond cost-per-episode comparisons between NPPs and physicians, the increased use of imaging over time has received attention because of increased aggregate imaging costs and population radiation exposure.^31^ Such concerns have led to efforts, such as Image Wisely, to limit injudicious imaging use that may be redundant, not clinically warranted, or wasteful.^32^ As such, appropriate imaging use is a broader policy issue. To the degree that NPPs in the ED use more imaging than physicians, their increased role in ED care may represent an opportunity for initiatives to ensure their judicious use of imaging. For example, we found that the 5.3% increase in imaging orders reflected both a 3.4% increase in the share of ED visits that had imaging and a 2.2% increase in the number of imaging studies for patients who had imaging. Accordingly, efforts to reduce variation in ordering practices in the ED need to address both when and what imaging is appropriate for specific clinical conditions. These efforts should be tailored to clinician type.^33^

This study found that increases in imaging ordering patterns associated with the presence of NPPs varied by modality (7.3% for CT, 3.2% for radiography, and 14.2% for all other modalities combined). Correspondingly, studies of ED imaging trends have shown substantially more growth in CT, ultrasound, and MRI than radiography.^4,8,10,11^ Given that in our study, 20.2%, 39.3%, and 4.4% of ED visits involved CT, radiography, and other imaging, respectively, it may be that higher variation is associated with less common but rapidly increasing use of CT, MRI, and ultrasound. Differences in imaging ordering practices by modality may inform efforts to reduce variation. Furthermore, greater imaging use was found across all diagnosis groups, ranging from 2.7% more for injury or poisoning to 11.7% more for neoplasms. Hence, differences in imaging ordering practices by diagnosis group may also inform variation reduction efforts.

The association of state NPP share with more imaging in the ED suggests variation in practice patterns of physicians compared with NPPs. To our knowledge, there is no literature on ED imaging ordering practice variation between physicians and NPPs. However, there are studies of ED imaging ordering practice variation among physicians. One single-site study found moderate variation,^34^ and another single-site study estimated that 1% of variation was due to physicians.^35^ More broadly, a variation study of chest CT in the diagnosis of suspected pulmonary embolism found that more-experienced physicians had lower imaging use and a higher diagnostic yield and that physicians who were board certified in emergency medicine had lower imaging use and the same diagnostic yield.^36^ Hence, experience and expertise appear to be associated with less imaging.

### Limitations

This study has some limitations. First, some ED visits billed by a physician may have been jointly managed (ie, done in consultation) with an NPP; hence, there is some potential error in determining the complete role of NPPs in ED imaging ordering patterns. To the degree that NPPs are associated with more imaging ordering than physicians, these jointly managed visits would result in an underestimate of the increase on imaging ordering patterns associated with the presence of NPPs. Second, given the limitations of claims data, we cannot fully control for clinical differences across ED visits, such as case mix severity, which would enable a direct comparison of imaging ordering practices between NPPs and physicians on a visit-by-visit basis. For this reason, we used the state-year NPP share as an instrument. Differences across state NPP share may be partly due to differences in NPP scopes of practice across states and over time, as well as variation across states and over time in how NPPs are used in EDs, incentive structures, and malpractice environments, all of which may influence imaging ordering patterns.

## Conclusions

In this cross-sectional study, we found that NPPs are associated with an increased likelihood of an ED visit involving imaging, and for ED visits with imaging, a greater number of imaging studies were performed per visit. Expanding the use of NPPs in the ED setting may improve patient access, but the associated increased use of imaging resources with associated cost and population and individual radiation dose must be considered. Furthermore, the presence of NPPs in the ED is associated with relatively more imaging for CT and other modalities (eg, MRI, ultrasound) than they are with radiography. Differences in imaging ordering practices by modality may inform efforts to reduce practice variation.

## Notes

Supplement. **eTable 1.** Two-Stage Model Results for Imaging Counts by Modality (All Emergency Department [ED] Visits) **eTable 2.** Two-Stage Model Results for Imaging Counts by Modality (Sensitivity Sample of Emergency Department [ED] Visits)
