# Supplementary material for: Association of State Share of Nonphysician Practitioners With Diagnostic Imaging Ordering Among Emergency Department Visits for Medicare Beneficiaries
Source: JAMA Netw Open. 2022 Nov 10;5(11):e2241297. doi: 10.1001/jamanetworkopen.2022.41297 (PMC9650604; doi:10.1001/jamanetworkopen.2022.41297)
Supplement: Supplement. — eTable 1. Two-Stage Model Results for Imaging Counts by Modality (All Emergency Department [ED] Visits) eTable 2. Two-Stage Model Results for Imaging Counts by Modality (Sensitivity Sample of Emergency Department [ED] Visits) [file jamanetwopen-e2241297-s001.pdf]

Supplemental Online Content

Christensen EW, Liu CM, Duszak R Jr, Hirsch JA, Swan TL, Rula EY. Association of state share of nonphysician practitioners with diagnostic imaging ordering among emergency department visits for Medicare beneficiaries. *JAMA Netw Open*. 2022;5(11):e2241297. doi:10.1001/jamanetworkopen.2022.41297

eTable 1. Two-Stage Model Results for Imaging Counts by Modality (All Emergency Department [ED] Visits)

eTable 2. Two-Stage Model Results for Imaging Counts by Modality (Sensitivity Sample of Emergency Department [ED] Visits)

This supplemental material has been provided by the authors to give readers additional information about their work.

© 2022 Christensen EW et al. *JAMA Network Open.*

eTable 1. Two-Stage Model Results for Imaging Counts by Modality (All Emergency Department [ED] Visits)

	**All Modalities**		**CT**		**Radiograph**		**Other**	
	**Stage 1**	**Stage 2**	**Stage 1**	**Stage 2**	**Stage 1**	**Stage 2**	**Stage 1**	**Stage 2**
State NPP share	0.628***	0.165***	0.767***	0.034***	0.504***	-0.002	1.171***	0.009
Age Group (Ref: 65-69)								
<65	-0.148***	-0.026***	-0.189***	0.004***	-0.132***	-0.003***	-0.079***	0.017***
70-74	0.028***	0.015***	0.055***	0.000	0.033***	0.005***	-0.003	0.002
75-79	0.036***	0.035***	0.098***	-0.001	0.058***	0.017***	-0.041***	0.004*
80-84	0.038***	0.055***	0.140***	-0.000	0.080***	0.031***	-0.107***	0.001
85-89	0.040***	0.078***	0.172***	0.001	0.100***	0.050***	-0.169***	-0.007***
90+	0.044***	0.102***	0.189***	0.014***	0.128***	0.072***	-0.272***	-0.025***
Female	0.106***	0.019***	0.073***	-0.017***	0.077***	0.033***	0.065***	0.003**
Race (Ref: white)								
Black	-0.090***	-0.021***	-0.163***	-0.032***	-0.019***	-0.010***	-0.056***	0.011***
Other	-0.073***	0.005***	-0.070***	-0.013***	-0.054***	0.012***	0.068***	0.017***
CCI group (Ref: 0)								
1	0.107***	0.002**	0.046***	0.000	0.114***	0.006***	-0.035***	-0.009***
2	0.117***	0.006***	0.031***	0.002**	0.139***	0.010***	-0.033***	-0.014***
3+	0.117***	0.013***	-0.043***	0.002**	0.200***	0.021***	-0.075***	-0.022***
Unknown	-0.030***	-0.001	-0.051***	-0.003	-0.007	0.002	-0.001	-0.011***
ED visit severity (Ref:1-3)								
4	1.035***	0.192***	1.748***	0.003*	0.563***	0.126***	1.404***	0.018 ***
5	1.116***	0.354***	2.062***	0.096***	0.764***	0.155***	1.514***	0.089 ***
Metro. residence	0.065***	0.038***	0.089***	0.023***	0.018***	0.018***	0.424***	0.040 ***
Patient had cancer	0.014***	-0.008***	0.048***	-0.004***	-0.039***	-0.012***	0.102***	0.006***
Patient died	-0.790***	0.080***	-0.555***	0.139***	-0.658***	0.049***	-0.533***	-0.045***
Patient admitted	-0.333***	0.109***	-0.131***	0.055***	-0.208***	0.051***	-0.053***	0.032***
In last 30 days, had claim for								
IP discharge	-0.066***	-0.024***	-0.175***	-0.020***	0.013***	-0.017***	-0.012**	-0.025***
ED visit	-0.182***	0.003***	-0.116***	0.012***	-0.163***	0.012***	-0.052***	0.002
Imaging	-0.052***	-0.002**	-0.066***	-0.002**	-0.044***	-0.001	0.100***	-0.004**
Non-imaging	0.107***	-0.018***	0.054***	-0.017***	0.085***	-0.012***	0.037***	-0.011***

Abbreviations: ED, emergency department; NPP, non-physician practitioner; CCI, Charlson Comorbidity Index; CT, computed tomography.
Note: Stage 1 is a logistic model for had imaging. Stage 2 is generalized linear model for the amount of imaging for visits with imaging. The model also controlled for diagnostic group, and year, but coefficients for these variables and the constant are not shown. *, **, and *** mean p<0.05, p<0.01, and p<0.001, respectively.

© 2022 Christensen EW et al. *JAMA Network Open.*

eTable 2. Two-Stage Model Results for Imaging Counts by Modality (Sensitivity Sample of Emergency Department [ED] Visits)

	All Modalities		CT		Radiograph		Other	
	Stage 1	Stage 2	Stage 1	Stage 2	Stage 1	Stage 2	Stage 1	Stage 2
State NPP share	0.663***	0.145***	0.826***	0.021	0.502***	-0.011	1.409***	-0.002
Age group (Ref: 65-69)								
<65	-0.170***	0.027***	-0.207***	0.006**	-0.147***	-0.003*	-0.107***	0.014***
70-74	0.047***	0.019***	0.069***	-0.002	0.054***	0.006***	0.018	0.004
75-79	0.065***	0.046***	0.131***	-0.005*	0.088***	0.025***	-0.017	0.000
80-84	0.082***	0.072***	0.190***	-0.009***	0.125***	0.045***	-0.083***	-0.001
85-89	0.080***	0.097***	0.231***	-0.011***	0.139***	0.069***	-0.170***	-0.015**
90+	0.089***	0.128***	0.259***	-0.004	0.170***	0.096***	-0.276***	-0.033***
Female	0.083***	0.013***	0.022***	-0.031***	0.069***	0.035***	0.088***	0.010***
Race (Ref: white)								
Black	-0.159***	-0.012***	-0.175***	-0.026***	-0.087***	-0.006***	-0.092***	0.017***
Other	-0.118***	0.011***	-0.072***	-0.007**	-0.102***	0.013***	0.066***	0.024***
CCI group (Ref: 0)								
1	0.207***	-0.004**	0.107***	-0.002	0.195***	0.003*	0.000	-0.008*
2	0.224***	-0.002	0.082***	-0.002	0.232***	0.005**	0.021*	-0.017***
3+	0.233***	0.005***	0.042***	-0.002	0.283***	0.013***	-0.012	-0.028***
Unknown	-0.038***	0.000	-0.040***	0.001	-0.029***	-0.001	0.012	0.000
ED visit severity (Ref:1-3)								
4	1.031***	0.215***	1.834***	0.012***	0.576***	0.143***	1.573***	0.032***
5	1.086***	0.402***	2.174***	0.115***	0.737***	0.187***	1.760***	0.109***
Metro. residence	0.054***	0.038***	0.079***	0.025***	0.013***	0.018***	0.408***	0.046***

Abbreviations: ED, emergency department; NPP, non-physician practitioner; CCI, Charlson Comorbidity Index; CT, computed tomography.
Note: The sensitivity sample was limited to ED visits for which the patient did not have cancer, was not admitted to the hospital, died the same day, or had any claim in the 30 days prior. Stage 1 is a logistic model for had imaging. Stage 2 is generalized linear model for the amount of imaging for visits with imaging. The model also controlled for diagnostic group, and year, but coefficients for these variables and the constant are not shown. *, **, and *** mean p<0.05, p<0.01, and p<0.001, respectively.

© 2022 Christensen EW et al. *JAMA Network Open.*
